# Is the efficacy of biological control against plant diseases likely to be more durable than that of chemical pesticides?

**DOI:** 10.3389/fpls.2015.00566

**Published:** 2015-07-27

**Authors:** Marc Bardin, Sakhr Ajouz, Morgane Comby, Miguel Lopez-Ferber, Benoît Graillot, Myriam Siegwart, Philippe C. Nicot

**Affiliations:** ^1^Plant Pathology Unit, Institut National de la Recherche Agronomique, UR407, Montfavet, France; ^2^Laboratoire de Génie de l’Environnement Industriel, Ecole des Mines d’Alès, Institut Mines-Telecom, Alès, France; ^3^Natural Plant Protection,Arysta LifeScience Group, Pau, France; ^4^Plantes et Systèmes de Culture Horticoles Unit, Institut National de la Recherche Agronomique, UR1115, Avignon, France

**Keywords:** biocontrol, durability, plant pathogen, resistance, efficacy

## Abstract

The durability of a control method for plant protection is defined as the persistence of its efficacy in space and time. It depends on (i) the selection pressure exerted by it on populations of plant pathogens and (ii) on the capacity of these pathogens to adapt to the control method. Erosion of effectiveness of conventional plant protection methods has been widely studied in the past. For example, apparition of resistance to chemical pesticides in plant pathogens or pests has been extensively documented. The durability of biological control has often been assumed to be higher than that of chemical control. Results concerning pest management in agricultural systems have shown that this assumption may not always be justified. Resistance of various pests to one or several toxins of *Bacillus thuringiensis* and apparition of resistance of the codling moth *Cydia pomonella* to the *C. pomonella* granulovirus have, for example, been described. In contrast with the situation for pests, the durability of biological control of plant diseases has hardly been studied and no scientific reports proving the loss of efficiency of biological control agents against plant pathogens in practice has been published so far. Knowledge concerning the possible erosion of effectiveness of biological control is essential to ensure a durable efficacy of biological control agents on target plant pathogens. This knowledge will result in identifying risk factors that can foster the selection of strains of plant pathogens resistant to biological control agents. It will also result in identifying types of biological control agents with lower risk of efficacy loss, i.e., modes of action of biological control agents that does not favor the selection of resistant isolates in natural populations of plant pathogens. An analysis of the scientific literature was then conducted to assess the potential for plant pathogens to become resistant to biological control agents.

## Introduction

The durability of a control method for plant protection is defined as the persistence of its efficacy in space and time. Erosion of effectiveness of conventional plant protection methods has been widely studied in the past. The durability of chemical control has for instance been studied because of the frequent and recurrent apparition of resistance to fungicides in major plant pathogenic fungal populations (Brent and Hollomon, 2007). The breakdown of varietal resistance, especially that conferred by major resistance genes, has also been widely studied for plant pathogens (McDonald and Linde, 2002). In contrast, the durability of biological control has long been assumed to be higher than that of chemical control (Holt and Hochberg, 1997). However, recent results concerning pest management in agricultural systems have shown that this assumption may not always be justified.

The most striking example may be the development of resistance to the most widely used bio-insecticide in the world. Resistance to one or several toxins produced by the bacterium *Bacillus thuringiensis* (*Bt*) has been described shortly after the market approval of products based on various strains of this bacterium. McGaughey was the first to observe resistance in the field to *Bt* toxins in the Indian meal moth *Plodia interpunctella*, a major post-harvest pest of grain (McGaughey, 1985; McGaughey and Johnson, 1992). Two other species have shown to present a resistance to *Bt*-formulation applied in the field: *Plutella xylostella* (Tabashnik, 1994) and *Trichoplusia ni* (Janmaat and Myers, 2003). Resistance of pests to other biocontrol agents has also been reported, one example is the codling moth *Cydia pomonella*. A granulovirus preparation has been used for 15 years as a bioinsecticide for its control and it is estimated that this product was applied to more than 100,000 ha of orchards in Europe and to two to three million ha worldwide, with a market steadily increasing due to its effectiveness and environmental safety (Eberle and Jehle, 2006; Asser-Kaiser et al., 2007). In 2004 insect populations with low susceptibility to the virus were detected for the first time in France, Italy, and Germany in pome fruit orchards (Fritsch et al., 2005; Eberle and Jehle, 2006; Sauphanor et al., 2006; Berling et al., 2009).

In contrast with the situation for pests, the durability of biological control of plant diseases has hardly been studied and no scientific paper proving the loss of efficiency of biocontrol agents against plant pathogens in practice has been published so far. This may be related to the limited use of biological control against plant diseases in practice until recently (Nicot et al., 2011a). A bibliographical study conducted in the framework of the European project ENDURE (European Network for Durable Exploitation of Crop Protection Strategies) established that despite the large amount of microorganisms potentially candidate for biological control (Nicot et al., 2011a), they are still few biocontrol agents registered against plant diseases in the European Union (Heilig et al., 2011).

However, several studies reported the inconsistency of efficacy of various biocontrol agents when introduced under commercial field conditions—being less effective or completely ineffective-even though their efficacy was very good in controlled conditions (Shtienberg and Elad, 1997; Guetsky et al., 2001; Mark et al., 2006; Nicot et al., 2011b). This variability of efficacy is generally attributed to climatic variations (temperature, humidity, radiation) encountered in field conditions, a lack of ecological competence (survival, colonization ability) of the biocontrol agent, intrinsic traits of the antagonistic microbe (variable production of required metabolites or enzymes) and/or an unstable quality of the formulated product (Elad and Stewart, 2004; Mark et al., 2006; Ruocco et al., 2011). However, reduction of efficacy in the field may also result from the diversity of sensitivity of plant pathogens to biocontrol agents, with the existence of less sensitive isolates in natural populations of plant pathogens.

The durability of biological control against plant pathogens may be related to specific traits of the plant pathogen such as genetic diversity and ability to evolve in response to a selection pressure. This is affected by population genetic processes including mutation, population size, recombination, gene flow and selection. This point was extensively studied to achieve durable plant disease resistance in agriculture (McDonald and Linde, 2002; McDonald, 2014). Thus, McDonald and Linde (2002) have hypothesized that populations of plant pathogens with high evolutionary potential are more likely to overcome a varietal resistance. The same assumption can be proposed for the development of resistance to biocontrol agents.

The durability of biological control against plant pathogens may also be related to the selection pressure exerted by the biocontrol agent. This selection pressure clearly depends on the extent of use of biocontrol agents in practice (surfaces treated, doses of application…). It may also depend on the specific mode of action of biocontrol agents. Various modes of action are involved in the protective effect of biocontrol agents against plant pathogens. Although the number of studies done on this subject is important, knowledge of the precise mode of action of biocontrol agents is still partial. However, it is generally considered that there are three main ways for a biocontrol agent to control a plant pathogen (Jacobsen, 2006; Alabouvette et al., 2009): first, by acting directly on the plant pathogen, through antibiosis, competition for nutrient or space, or parasitism; secondly by interfering with the mechanisms of pathogenesis of the plant pathogen, and thirdly by modifying the interaction of the plant pathogen with its plant host for instance through the induction of local or systemic acquired resistance. These modes of action are not incompatible, they can instead be complementary and a single species or a single strain of a biocontrol agent may act with several of these modes of action (Janisiewicz and Korsten, 2002). A given biocontrol agent may therefore operate through several mechanisms potentially expressed successively, simultaneously or synergistically and possibly depending on the environmental conditions encountered. Nevertheless, it is not yet clear if biocontrol agents have a dominant mode of action and under what conditions they switch from a mode of action to another. Even though all biocontrol agents should create selection pressure on target populations of plant pathogens once treatments are applied in the field, some modes of action may present a clear opportunity for pathogens to evolve resistance.

The main objective of this review is to assess the potential for plant pathogens to become resistant to biocontrol agents. To this end, an analysis of the scientific literature was conducted and scientific papers reporting the diversity of efficacy of biocontrol agents toward plant pathogens or those describing the ability of plant pathogens to produce natural mutants with reduced susceptibility under the selection pressure exerted by biocontrol agents were analyzed. The link between a potential loss of efficacy of a biocontrol agent and its mode of action was carefully explored.

## Survey of the Scientific Literature Regarding the Durability of Biological Control Against Plant Diseases

Identifying publications focused on this topic proved to be a challenging task. A survey of the Web of Science database between 1973 and the end of 2014, using the keyword combination [(biological control OR biocontrol OR antagonist*) AND ((pathogen OR disease) AND plant)], yielded 7872 references on biological control against plant diseases. Entering keywords describing the diversity of efficacy of biocontrol agents toward plant pathogens, i.e., [(divers* OR variab* OR variation) AND (resist* OR toleran* OR susceptib* OR sensitiv* OR insensitiv* OR defense)], allowed to refine this survey to 593 references. However, the analysis of this subset yielded only six relevant references. A second subset was generated from the initial 7872 references by entering keywords describing the durability of efficacy of biocontrol agents toward plant pathogens, i.e., (durability OR durable OR sustainable OR sustainability). Among the 274 references obtained, only one was relevant. Efforts to improve the keyword combinations failed to yield any additional references, a situation presumably reflecting the scarcity of studies specifically dedicated to this topic. The set of seven references was then complemented with studies including a comparison of biocontrol efficacy for two or more strains of a given plant pathogen. Attempts to automate this search with keywords failed, probably due to the fact that such comparisons were not the focus of these studies. Eventually, additional references were obtained through direct consultation with scientists implicated in biocontrol of plant pathogens. It is thus likely that the final set of 30 references analyzed in the present review may not be exhaustive, but it already provided quite illustrative examples (Table 1). The vast majority of the publications provided information on the diversity of sensitivity to biocontrol agents or plant extracts among isolates of a given plant pathogen and only three publications concerned the ability of plant pathogens to adapt to biological control. Biocontrol agents described in the references have been classified according to their modes of action (basically antibiosis, hyperparasitism, competition, and induced resistance).

**TABLE 1:** **Scientific papers published between 1973 and 2014 exploring the diversity of sensitivity of biological control against plant diseases analyzed in the review**.

	Number of references (Number of species*)	
Plant pathogens		
Bacteria	4	(4)
Fungi	19	(13)
Oomycetes	5	(2)
Biocontrol agents		
Virus	3	(2)
Bacteria	14	(>18)
Fungi	8	(>8)
Plant extract	3	(3)
Mode of action of biocontrol agents		
Antibiosis	16	
Hyperparasitism	6	
Combination of modes of action	6	

*Number of microbial or botanical species evaluated for the plant pathogens and the biocontrol agents.

## Diversity of Sensitivity of Plant Pathogens to Biocontrol Agents

Possible loss of efficacy of a biocontrol agent could result from the pre-existence of isolates with low susceptibility in natural populations of plant pathogens. Published studies that evaluated the diversity of sensitivity to biocontrol agents within a plant pathogen species were examined separately, according to the mode of action of the biocontrol agent or to the type of test realized.

### *In Vitro* Variation of Sensitivity of Plant Pathogens to Antimicrobial Compounds Produced by Biocontrol Agents

Studies highlighting the variation in sensitivity to antimicrobial compounds produced by antagonistic microorganisms are listed in Table 2. Six different antimicrobial compounds have been tested against five species of plant pathogens. For a given species, the number of tested isolates ranged from 3 to 204.

**TABLE 2:** **Examples highlighting the variation in sensitivity of plant pathogens to antimicrobial compounds produced by biological control agents**.

**Compounds evaluated^1^**	**BCA^2^**	**Plant pathogen**	**Number of isolates**	**Main results**	**Reference**
2,4-DAPG	*Pseudomonas* spp.	*Gaeumannomyces graminis* var. *tritici*	66	Diverse levels of sensitivity; some isolates not controlled in the rhizosphere of wheat.	Mazzola et al. (1995)
2,4-DAPG	*Pseudomonas fluorescens*	*Fusarium oxysporum*	117	17% of isolates naturally tolerant (RF^3^ ≈ 10).	Schouten et al. (2004)
2,4-DAPG	*Pseudomonas* spp.	*Pythium* spp.	13 species	*P. volutum* is the most sensitive, *P. deliense* is the most resistant; various isolates of *P. ultimum* var. *sporangiiferum* differ in sensitivity.	De Souza et al. (2003)
PCA	*Pseudomonas* spp.	*Gaeumannomyces graminis* var. *tritici*	66	Diverse levels of sensitivity; some isolates not controlled in the rhizosphere of wheat.	Mazzola et al. (1995)
PCA	*Pseudomonas* spp.	*Pythium* spp.	9 species	*P. ultimum* var. *sporangiiferum* has the lowest sensitivity.	Gurusiddaiah et al. (1986)
Kanosamine	*Bacillus cereus*	*Pythium* spp.	3 species	*P. aphanidermatum* and *P. torulosum* are less sensitive.	Milner et al. (1996)
Lipopeptide Massetolide A	*Pseudomonas fluorescens* SS101	*Pythium* spp.	8 species	*P. heterothallicum*, *P. rostratum*, *P. ultimum* have a greater tolerance.	Mazzola et al. (2007)
Gliotoxin	*Gliocladium virens*	*Rhizoctonia solani*	18	Some Anastomosis Groups less sensitive than others.	Jones and Pettit (1987)
Pyrrolnitrin	*Pseudomonas chlororaphis*	*Botrytis cinerea*	204	Diverse levels of sensitivity (RF = 8.4 for the most resistant isolates); isolates controlled by the BCA on tomato stem.	Ajouz et al. (2011b)

^1^Antimicrobial compounds produced by biocontrol agents: 2,4-DAPG, 2,4-diacetylphloroglucinol; PCA, phenazine-1-carboxylic acid. ^2^BCA, biocontrol agent. ^3^RF, resistance factor.

Among 66 isolates of *Gaeumannomyces graminis* var. *tritici*, the causal agent of take-all of wheat, diverse levels of sensitivity were observed toward phenazine-1-acide carboxylique (PCA) and 2,4-diacetylphloroglucinol (2,4-DAPG), two compounds produced by antagonistic strains of fluorescent *Pseudomonas* spp. present in the wheat rhizosphere (Mazzola et al., 1995). One isolate of *G. graminis* var. *tritici*, was resistant to both antibiotics and could not be controlled effectively by the biocontrol agent in the rhizosphere of wheat plants. This led Mazzola et al. (1995) to speculate that massive use of these antibiotic-producing biocontrol agents might eventually result in the selection of *G. graminis* var. *tritici* populations resistant to these biocontrol agents. Diversity in the sensitivity to 2,4-DAPG was also found in a collection of 117 isolates of the fungal plant pathogen *Fusarium oxysporum*, with approximately 17% of the isolates being naturally tolerant (Schouten et al., 2004). The least sensitive isolates of *F. oxysporum* were able to withstand a 10-fold higher concentration of 2,4-DAPG than the lethal concentration used for isolates with the highest level of sensitivity. No correlation was found between the geographic origin of strains and their tolerance to 2,4-DAPG, suggesting that this tolerance was widely distributed worldwide among different subpopulations of *F. oxysporum*. A wide range of sensitivity was also reported in *Botrytis cinerea* to pyrrolnitrin, an antimicrobial compound produced by several antagonistic bacterial species including *Serratia plymuthica* and various Pseudomonads (Ajouz et al., 2011b). Among 204 isolates of *B. cinerea*, differing in their geographic origin and host of isolation, several had a significantly reduced sensitivity to pyrrolnitrin, with a resistance factor reaching 8.4-fold compared to the most sensitive isolates. These results raised the question of whether this pre-existing moderate level of resistance could foster the acquisition of a higher level of resistance to pyrrolnitrin through selective pressure, should the use of pyrrolnitrin-producing biocontrol agents become widespread in the field.

This diversity of sensitivity to antimicrobial compounds produced by biocontrol agents was also observed among different anastomosis groups within a species or different species of plant pathogens that could occur and can infect plants at the same time. For instance, variation in sensitivity among 18 isolates of *Rhizoctonia solani* to the antibiotic gliotoxin (produced by the biocontrol fungus *Gliocladium virens*) was observed, with isolates of some anastomosis groups being less sensitive than others (Jones and Pettit, 1987). This was also the case for individual isolates of several closely related species of *Pythium*, commonly found together in symptoms developed by this complex of soilborne oomycetes. Differences of sensitivity among species of this complex were reported for PCA, *P. ultimum* var. *sporangiferum* being the least sensitive (Gurusiddaiah et al., 1986), for kanosamine (an antibiotic synthesized by the bacterium *Bacillus cereus*), *P. medicaginis* being less sensitive than *P. aphanidermatum* and *P. torulosum* (Milner et al., 1996), for the cyclic lipopeptide massetolide, *P. heterothallicum*, *P. rostratum*, and *P. ultimum* var. *ultimum* being the least sensitive of eight *Pythium* species (Mazzola et al., 2007), and for 2,4-DAPG, *P. volutum* being the most sensitive and *P. deliense* the least sensitive of 13 species (De Souza et al., 2003). One striking aspect of this latter study is that various types of asexual propagules of *P. ultimum* var. *sporangiiferum* (mycelium, zoosporangia, zoospore cysts, and zoospores) differed considerably in their sensitivity to 2,4-DAPG. The difference of susceptibility among *Pythium* species to various natural toxic compounds produced by biocontrol agents and the fact that several of these species could occur at the same time in a similar specific place may impact the success or at least the regularity of the effectiveness of biological control conferred by these antibiotic-producing bacterial strains.

### Variation in Sensitivity of Plant Pathogens to Biocontrol Agents or Plant Extracts Having a Single Mode of Action

Several studies have been conducted on the antibiotic-producing biocontrol agents themselves, and not only on the antimicrobial compounds involved in their effectiveness (Table 3). A well documented case concerns the control of bacterial plant pathogen *Agrobacterium tumefaciens*, the causal agent of crown gall on many dicotyledonous plants, by biocontrol agent *A. rhizogenes* strain K84, which produces the antibiotic agrocin 84 (Moore and Warren, 1979). Among 65 strains of *A. tumefaciens*, all those belonging to biotype three were resistant to the biocontrol agent *in vitro*, whereas many of biotype 1 and biotype 2 strains were susceptible (VAN Zyl et al., 1986).

**Table 3:** **Examples highlighting the variation in sensitivity of plant pathogens to biological control agents or plant extracts described as having a simple mode of action**.

**Modes of action of BCA**	**BCA/plant extract**	**Plant pathogen**	**Number of isolates**	**Main results**	**Reference**
Antibiosis	*Agrobacterium rhizogenes*	*Agrobacterium tumefaciens*	65	Biotype 3 are resistant to the antibiotic agrocin 84.	VAN Zyl et al. (1986)
	*B. subtilis*, *Serratia plymuthica*, *P. putida*, *Streptomyces setonii*	*Rhizoctonia solani*	8	Diverse levels of sensitivity (dual culture assay *in vitro*).	Faltin et al. (2004)
	*Bacillus mojavensis*	*Fusarium verticillioides*	3	Diversity in percent of lesion reduction in maize.	Bacon and Hinton (2011)
	4 endophytes of elm + 2 *Trichoderma* strains	*Ophiostoma novo-ulmi*	6	*In vitro* growth inhibition less marked in some strains of *O. novo-ulmi*.	Diaz et al. (2013)
	15 fungal endophyte species	*Gremmeniella abietina*	4	Diverse levels of sensitivity (dual culture assay *in vitro*).	Santamaria et al. (2007)
	20 strains of *Streptomyces* spp.	*Streptomyces scabies*	15	Diverse levels of sensitivity; 1 isolate resistant to 16 BCA strains.	Otto-Hanson et al. (2013)
	3 strains of *Streptomyces* spp.	*Botrytis cinerea*	41	*In vitro* growth inhibition less marked in some strains of *B. cinerea*.	Boukaew et al. (2015)
	Aqueous extract of *Cuscuta pedicellata* L.	*Xanthomonas campestris*	different pathovars	*In vitro* variation in susceptibility among pathovars of *X. campestris*.	Ali et al. (2013)
Hyperparasitism	*Cryphonectria* hypovirus 1 (CHV-1)	*Cryphonectria parasitica*	6 8	Difference in tolerance; small magnitude of tolerance.	Peever et al. (2000), Bryner and Rigling (2012)
	5 bacteriophages	*Erwinia amylovora*	52	23 strains exhibit resistance to at least one phage.	Schnabel and Jones (2001)
	*Acremonium strictum*	*Helminthosporium solani*	4	Diverse levels of sensitivity.	Rivera-Varas et al. (2007)
	*Coniothyrium minitans*	*Sclerotinia sclerotiorum S. trifoliorum*	34 9	Considerable variation in the rate and extend of infection by *Coniothyrium*.	Turner and Tribe (1976)
	*Coniothyrium minitans*	*Sclerotinia sclerotiorum*	3	1 strains less susceptible to infection.	Huang et al. (2011)

Variability was also observed with biological control of potato scab, caused by *Streptomyces scabies*. Otto-Hanson et al. (2013) reported that *in vitro* growth inhibition of *S. scabies*, using antibiotic-producing isolates of *Streptomyces* sp., varied depending on the isolates of the plant pathogen. Using 15 isolates of *S. scabies* and 19 antagonistic strains of *Streptomyces* sp, they showed that there was significant variation among pathogen isolates in their susceptibility to inhibition, and one isolate exhibited low susceptibility and was inhibited by only four of the antagonistic strains. This illustrated that there was a high level of diversity both among the isolates of the pathogen and among the antagonists. Interestingly, only one of the 19 antagonistic isolates was able to inhibit the growth of all 15 isolates of *S. scabies*. In the same vein, using a dual-*in vitro* culture technique, Boukaew et al. (2015) observed that among 41 strains of *B. cinerea*, only 31, 31, and 25 were completely inhibited by strains RM-1-138, RL-1-178, and SS-2-243 of *Streptomyces* spp., respectively.

Some level of variability in the sensitivity to biocontrol agents has also been encountered in other studies involving fewer strains of plant pathogens. This was the case for example, among eight isolates of *Rhizoctonia solani* examinated for their sensitivity to antibiotic-producing bacterial strains of *Bacillus subtilis*, *Serratia plymuthica*, *Pseudomonas putida*, and *Streptomyces setonii* (Faltin et al., 2004) and among six strains of *Ophiostoma novo-ulmi* evaluated for their sensitivity to four endophytic fungi of elm and two *Trichoderma* strains (Diaz et al., 2013). Finally, the *in vitro* susceptibility of *Xanthomonas campestris* to aqueous extracts of the plant species *Cuscuta pedicellata* was shown to vary according to the pathovar of the bacterium (Ali et al., 2013).

All these examples clearly highlight the challenge one may face for the development of a single-strain biocontrol agent with wide effectiveness against diverse populations of plant pathogens.

### Variation in Sensitivity of Plant Pathogens to Hyperparasites

Several studies have explored the diversity of sensitivity to hyperparasitic biocontrol agents (Table 3). The best documented cases involve viral (hyper)parasites of plant pathogens. One success story of biological control against plant diseases concerns the *Cryphonectria* hypovirus 1 (CHV-1) that hyperparasitizes the fungus *Cryphonectria parasitica* and reduces its pathogenicity on chestnut trees, resulting in hypovirulent isolates of the fungus (Milgroom and Cortesi, 2004). Infection with CHV-1 is persistent and does not kill *C. parasitica*. However, it inhibits its sexual reproduction and strongly attenuates the growth and conidial sporulation of the fungus. The resulting hypovirulence provides adequate control of chesnut blight in Europe and in some locations in the United States, with the exception of eastern North America. Differences in tolerance to CHV-1 in different populations of *C. parasitica* were detected (Peever et al., 2000; Bryner and Rigling, 2012). Even thought the magnitude of tolerance to the hypovirus is relatively small, these results suggest that there is a potential for future evolution of this character in the natural populations of the plant pathogenic fungus. Evidence that different strains of bacteria can present a diversity of sensitivity to bacteriophages was also proposed. In order to control fire blight caused by the bacterium *Erwinia amylovora*, bacteriophages were tested as biocontrol agents (Schnabel and Jones, 2001). The ability of five phages to infect 52 strains of *E. amylovora* varied dramatically. Only 22 of 52 strains were sensitive to all five phages, and 23 strains exhibited resistance to more than one phage.

Other studies concern fungal hyperparasites. *Coniothyrium minitans* is a hyperparasite of sclerotia produced by various pathogenic fungal species including *Sclerotinia* spp. (Gerlagh et al., 1996; Whipps et al., 2008). The susceptibility of *Sclerotinia* spp. to *C. minitans* was evaluated on 34 strains of *S. sclerotiorum* and nine strains of *S. trifoliorum*, revealing considerable variation in the rate and extend of sclerotial infection by the mycoparasite (Turner and Tribe, 1976). More recently, Huang et al. (2011) suggested that reduced susceptibility to sclerotial infection by *C. minitans* may be related to the production of oxalate by strains of *S. sclerotiorum*. Diversity in susceptibility was also observed for four strains of the plant pathogenic fungus *Helminthosporium solani* to the mycoparasite *Acremonium strictum*, with ranges of *in vitro* inhibition of 35–65%, 43–53%, and 32–40%, for sporulation, spore germination and mycelial growth of the plant pathogen, respectively (Rivera-Varas et al., 2007).

### Variation in Sensitivity of Plant Pathogens to Biocontrol Agents or Plant Extracts Combining Several Modes of Action

For many biocontrol agents, several modes of action have been identified and are believed to play a role in their protective effect against plant pathogens. A few studies have examined their efficacy toward different isolates of plant pathogens and revealed some level of variability (Table 4).

**Table 4:** **Examples highlighting the variation in sensitivity of plant pathogens to biological control agents or plant extracts having several modes of action**.

Mode of action*	**BCA/plant extract**	**Plant pathogen**	**Number of isolates**	**Main results**	**Reference**
Antibiosis + competition	*Rhodotorula glutinis* PM4	*Botrytis cinerea*	29	Variable susceptibility; some isolates totally resistant; no correlation with aggressiveness of *B. cinerea*.	Buck and Jeffers (2004)
Antibiosis + CWDE + IR	*Bacillus subtilis* QST713	*Botrytis cinerea*	20	Wide range of sensitivity; differences of efficacy between tomato and lettuce leaves; no correlation with aggressiveness of *B. cinerea*.	Bardin et al. (2013a)
Direct effect + IR	Plant extract from rhubarb	*Podosphaera xanthii*	162	Limited amount of diversity.	Yang et al. (2008)
	Plant extract from rhubarb	*Pseudoperonospora cubensis*	116	Limited amount of diversity.	Yang et al. (2008)
	Plant extract from giant knotweed	*Podosphaera xanthii*	52	Wide range of sensitivity, some isolates able to sporulate.	Bardin et al. (2013c)
	Plant extract from giant knotweed	*Golovinomyces cichoracearum*	5	Wide range of sensitivity, some isolates able to sporulate.	Bardin et al. (2013c)
Complex mode of action	*Trichoderma harzianum* strain T22	*Calonectria pauciramosa*	20	Ability to product microsclerotia and effect in controlling infection highly variable among isolates of *C. pauciramosa*.	Vitale et al. (2012)
Competition + other indirect effects?	*Microdochium dimerum*	*Botrytis cinerea*	41	High level of efficacy at recommended dose; Variable susceptibility at low dose: correlation of protection and aggressiveness of *B. cinerea*.	Bardin et al. (2013b)

*IR, induced plant resistance; competition, competition for nutrient; CWDE, cell-wall degrading enzyme.

For microbial biocontrol agents, this is the case for instance for the yeast *Rhodotorula glutinis* PM4, which was reported to inhibit *B. cinerea* both by competing for nutrients and by producing the toxic compound rhodotorulic acid (Sansone et al., 2005). In a study comparing 29 isolates of *B. cinerea* a wide range of susceptibility to the effect of the biocontrol agent was encountered, with several isolates showing complete resistance and producing symptoms (Buck and Jeffers, 2004). No obvious correlation between the relative aggressiveness of *B. cinerea* and the effectiveness of *R. glutinis* PM4 was observed. *B. cinerea* was also reported to display a wide range of susceptibility toward the protective effect of other beneficial microbes. One is *Bacillus subtilis* QST713, a biocontrol agent presumed to operate through various modes of action such as antibiosis, competition, hyperparasitism, and induction of resistance (Paulitz and Belanger, 2001; Lahlali et al., 2013). In a study comparing 20 strains of *B. cinerea* (differing in their geographic origin, host of isolation and level of aggressiveness), the protective efficacy of *B. subtilis* QST713 was found to range from 40 to 86% on tomato leaves and from 0 to 80% on lettuce leaves (Bardin et al., 2013a). Another study concerns strain L13 of *Fusarium sp.*, which was shown to protect pruning wounds of tomato against *B. cinerea* (Bardin et al., 2008). A very high level of efficacy was observed against 41 isolates of *B. cinerea* when the biocontrol agent was used at the recommended dose of 10^7^ spores/ml (Bardin et al., 2013b). However, at a 10-fold reduced dose of application the diversity of sensitivity of *B. cinerea* to this biocontrol agent was high. A correlation was noticed between the level of aggressiveness of *B. cinerea* and the level of protection provided by the biocontrol agent (Bardin et al., 2013b). This correlation is probably the consequence of the presumed mode of action of this biocontrol agent, which is not based on a direct effect but more probably linked to a competition for nutrients and space. Other examples concern species of one of the most studied genera of microbial biocontrol agent, *Trichoderma*. The effect of *Trichoderma* species on plant pathogens have been shown to involve several modes of action (Elad, 2000; Howell, 2003; Harman, 2006). In a study involving strain T22 of *T. harzianum*, Vitale et al. (2012) observed that its protective effect was highly variable among 20 isolates of the fungal plant pathogen *Calonectria pauciramosa* and that, in presence of the biocontrol agent, some isolates conserve the ability to produce about as much microsclerotia as the control without biocontrol agent.

Few studies have evaluated the diversity of susceptibility of plant pathogens to plant extracts having a complex mode of action (Table 4). One example is an extract from roots of Chinese rhubarb (*Rheum officinale* Baill.), which was shown to control powdery mildew of barley mainly through induced resistance and especially by enhancing expression of leaf-specific thionin in leaves (Ma et al., 2010). In a study to establish baselines of sensitivity for pathogens of cucumbers, tests on detached cotyledons with 162 isolates of *Podosphaera xanthii* (syn. *Sphaerotheca fuliginea*) and on leaf disks for 116 isolates of *Pseudoperonospora cubensis* revealed limited diversity among isolates of either species (Yang et al., 2008). Another example concerns leaf extracts of the giant knotweed *Fallopia (Reynoutria) sachalinensis*, whose mode of action is generally associated with plant-induced resistance (Daayf et al., 1997; Fofana et al., 2002), but which is also known to have a direct inhibitory effect on spore germination for different powdery mildew fungi, including *Blumeria graminis* (Randoux et al., 2006), *Oidium neolycopersici*, *Leveillula taurica*, or *Golovinomyces cichoracearum* (Table 5). A wide range of susceptibility was observed among 52 isolates of *P. xanthii* and five isolates of *Golovinomyces cichoracearum* on detached melon leaf disks treated with a commercial preparation of the extract (Bardin et al., 2013c). Sporulation by some isolates was observed on the leaf disks, suggesting that use of this plant extract on a commercial scale could possibly lead to the selection of resistant isolates.

**Table 5:** **Effect of the formulated plant extract from *Fallopia (Reynoutria) sachalinensis* (Milsana^®^) applied at different concentrations on the spore germination of three different powdery mildew species on solid medium (10 g/L agar), 72 h after inoculation**.

**Concentration of formulated plant extract**	**Spore germination rate (%)**		
	**Control**	**2.5 mL/L**	**2500 mL/L**
*Oidium neolycopersici*	81 ± 9	9 ± 1	0
*Leveillula taurica*	60 ± 8	13 ± 2	0
*Golovinomyces cichoracearum*	60 ± 9	42 ± 9	0

Results are the mean of three independent experiments. Each mean is associated with its standard error.

Biocontrol agents with multiple modes of action are commonly considered as presenting no, or very limited risk for the development of resistance by plant pathogens. Although the number of studies on this topic is still scant, this concept may be need to considered with caution as the examples shown above clearly demonstrate that plant pathogens can display a high level of diversity in their sensitivity to such biocontrol agents.

## Capacity of Plant Pathogens to Adapt to Biological Control

Besides existing diversity in susceptibility of plant pathogens to biocontrol agents, another concern could be that resistance would develop through adaptation under selection pressure, following the generalized use of a biological control method in the field, as has already occurred for various pathogens with certain fungicides. The estimation of this potential risk can be achieved through experimental evolution studies with the production of successive generations of the pathogens under selection pressure, as commonly carried out to evaluate the durability of efficacy of antimicrobial compounds in human pathology (Cowen et al., 2002), or to assess the capacity of plant pathogens to adapt to fungicides (Brent and Hollomon, 1998). One of the very few studies of this type conducted on biocontrol agents of plant diseases was carried out with *Bacillus subtilis* CL27, a bacterium producing three different antifungal compounds, two of which were effective *in vitro* against *B. cinerea* (Li and Leifert, 1994; Leifert et al., 1995). Following eight successive treatments on plants of *Astilbe hybrida*, the protective effect of the biocontrol agent against *B. cinerea* strongly decreased and control became totally ineffective after the 10th treatment (Li and Leifert, 1994). In another experimental evolution study, Ajouz et al. (2010) observed the acquisition of resistance by five isolates of *B. cinerea* to pyrrolnitrin. This buildup of resistance was correlated *in vitro* with a reduced inhibitory effect of the pyrrolnitrin-producing biocontrol strain ChPhzS24 of *P. chlororaphis*. For all isolates of *B. cinerea*, the adaptation was observed after 12 successive generations produced under selection pressure *in vitro*. The first 10 generations were grown on nutrient medium containing a concentration of pyrrolnitrin of 10 μg/L, which corresponded to an inhibition of mycelial growth comprised between 56 and 98% depending on the isolates. For these 10 generations, no major change in sensitivity to pyrrolnitrin was observed. In contrast, the production of additional generations on media containing higher doses of pyrrolnitrin resulted in the rapid development of variants with a high level of resistance to the antibiotic (RF > 1000). The same results were observed for all five strains of *B. cinerea*, regardless of their initial phenotype of sensitivity to the antibiotic (Ajouz et al., 2010). Interestingly, a similar experiment conducted with the same strains of *B. cinerea* on media containing the fungicide iprodione also led to buildup of resistance to iprodione and to pyrrolnitrin and to the pyrrolnitrin-producing biocontrol agent (Fillinger et al., 2012), suggesting that the use of chemical methods in parallel or in combination with biological control may have an impact on the durability of the efficacy of certain biocontrol agents.

In contrast to the situation with *B. cinerea*, no build up of resistance was observed after 15 generations for two biotrophic fungi (the powdery mildew fungus *P. xanthii* and the downy mildew oomycete *P. cubensis*) in an experimental evolution study on melon leaves treated with an extract from roots of Chinese rhubarb (Yang et al., 2008). This absence of evolution might be due to a complex mode of action of the plant extract, known to include both a direct antimicrobial effect and induced resistance (Ma et al., 2010). However, it would be interesting to assess if the production of additional generations of *P. xanthii* and *P. cubensis* on melon leaves treated with higher concentrations of the plant extract would result in the sudden build up of resistance as observed for *B. cinerea* by Ajouz et al. (2010). Interestingly, the high level of resistance to pyrrolnitrin attained by the last generations of *B. cinerea* in these experiments did not confer complete resistance to the effect of *P. chlororaphis* ChPhzS24 (Ajouz et al., 2010), a situation compatible with the report that other antibiotics, in addition to pyrrolnitrin, are produced by this strain (Schoonbeek et al., 2002). Finally, the buildup of resistance to pyrrolnitrin was associated with an important fitness cost for *B. cinerea*, with a significant reduction in mycelial growth, sporulation and ability to colonize plant tissues observed for the five isolates under study (Ajouz et al., 2010, 2011a).

To our knowledge, the three experimental evolution studies described here are the only examples illustrating the potential of plant pathogens to adapt to the effect of biocontrol agents. In two of these studies, the fungal pathogen (*B. cinerea*) was able to rapidly adapt to the effect of the antibiotic-producing biocontrol agents tested. However, in the case of the pyrrolnitrin-producing biocontrol agent, a detrimental effect for the plant pathogen of the resistance to pyrrolnitrin would limit the risk of complete loss of efficacy. Based on the study with physcion, it is likely that this plant extract will have a low to moderate risk for resistance development of powdery mildew and downy mildew of cucurbits. Additional studies with other biocontrol agents having different modes of action would be necessary to infer a possible link between the durability of efficacy of a biocontrol agent and its mode of action. Similarly, additional studies with other plant pathogens would allow to generate hypotheses about a possible link between the durability of biological control and specific traits related to the plant pathogen.

## Mechanisms Potentially Involved in the Resistance to Biocontrol Agents

Although only few studies have addressed the existence of resistance toward biocontrol agents among differents isolates of plant pathogens, mechanisms of resistance to toxic compounds produced by microorganisms or plants have been widely studied and extensively reviewed by several authors (Duffy et al., 2003; Raaijmakers et al., 2009). In this chapter we will present the main mechanisms of resistance of plant pathogens to potentially toxic compounds that can be directly or indirectly involved in the mechanisms of action of biocontrol agents (antibiosis, induced plant resistance, hyperparasitism). We will also present some studies that concern the tolerance of plant pathogens to nutritive stress which correspond to the mode of action of competition for nutrients.

### Resistance to Compounds Produced by Micro-Organisms

Antimicrobial compounds play a major role in the suppression of plant pathogens by antagonistic microorganisms, especially in the soil (Raaijmakers et al., 2002). Plant pathogenic fungi, oomycetes and bacteria have developed a range of strategies to tolerate or resist the deleterious effects of such compounds. These strategies involve mechanisms such as active efflux, degradation of antimicrobial compounds, and interference with the regulation and biosynthesis of antimicrobial metabolites produced by antagonistic microorganisms (reviewed in Duffy et al., 2003).

#### Excretion

As a first line of defense against toxic compounds, microbes possess ABC (ATP-binding cassette), or MSF (major facilitator superfamily) transporters. These membrane proteins are able to transport a wide range of compounds and can evacuate outside of the cells diverse toxic metabolites. The tolerance conferred by these transporters is quite low but it is significant because it provides time for pathogens to activate some mechanisms of detoxification (Schouten et al., 2008). ABC transporters are more often involved in the protection of fungi against antifungal compounds than MSF transporters are (De Waard et al., 2006). The role of efflux pumps has been implicated in the resistance of *B. cinerea* to antibiotic compounds produced by several potential biocontrol agents in the genus *Pseudomonas* (Schoonbeek et al., 2002). Several antibiotics such as phenazines (PCN) were shown to induce the expression of BcatrB, a gene coding for an ABC transporter, providing the first demonstration of the role of ABC transporters in microbial interactions, especially to protect a plant pathogen against an antibiotic produced by a biocontrol agent. ABC transporters have been shown to affect the sensitivity of fungi to various natural toxic metabolites and have been implicated in cases of multi-drug resistance (De Waard et al., 2006). Multidrug resistance (MDR) is emerging in the field among phytopathogenic fungi maintained under strong selection pressures and leads to the development of isolates resistant to fungicides belonging to different chemical families (Kretschmer et al., 2009). Ajouz et al. (2011b) observed that isolates of *B. cinerea* collected in the field and less sensitive to pyrrolnitrin were all multi-drug resistant isolates. In the plant pathogenic bacterium *E. amylovora*, a membrane transporter (NorM) was also shown to provide resistance to toxins produced by antagonistic bacteria *P. fluorescens* and *Pantoea agglomerans* (Burse et al., 2004). Therefore, these transporters could be a major mechanism of resistance to antibiotics-producing biocontrol agents that could be possibly combined with resistance to fungicides.

#### Metabolization

Other mechanisms have been involved in the resistance of plant pathogens to biocontrol agents acting through antibiosis. For example, exposure of *Mycosphaerella graminicola* to a sublethal dose of 1-hydroxyphenazine, an antibiotic produced by the biocontrol bacterium *Pseudomonas*, led to increased production by the fungus of catalases, peroxidases, and superoxide-dismutases and an increase in melanin synthesis, allowing the degradation of the antibiotic and the protection of the fungus (Levy et al., 1992). In another study using the bacterium *P. fluorescens*, *Rhizoctonia solani* resulted in the synthesis by the fungus of laccases in response to the effects of bacterial metabolites (Crowe and Olsson, 2001). These laccases render fungal cell walls less permeable to toxic compounds and can detoxify antifungal compounds produced by the bacterium.

#### Biosynthesis Inhibition

Interference with the biosynthesis of antimicrobial compounds can also be a means for plant pathogens to be resistant to biocontrol agents. Fusaric acid, a toxin produced by the plant pathogenic fungus *F. oxysporum*, represses the production of the antimicrobial 2,4-DAPG within the biocontrol strain *P. fluorescens* CHA0 (Notz et al., 2002). This repressive effect may affect the performance of biocontrol bacteria that are sensitive to fusaric acid. A plant pathogen can also reduce the antibiotic production of a biocontrol agent, by altering its chemical environment. For example the plant pathogenic fungus *Gaeumannomyces graminis* var *tritici* acidifies the environment, thus disturbing the activity of the antagonistic bacterium *P. fluorescens* by significantly decreasing its effectiveness (Ownley et al., 1992).

Finally, natural plasmid transfer between bacteria was also shown to provide a possible mechanism for plant pathogens to become resistant to biocontrol agents. This was the case for example for the soilborne bacterium *A. tumefaciens* which became resistant to biocontrol agent *A. radiobacter* K84 by acquiring its plasmid pAgK84 (Stockwell et al., 1996). The resistance resulted from the fact that this plasmid carries both the gene coding for the production of agrocin 84, the antibiotic responsible for the activity of the biocontrol agent, and the gene involved in the resistance to this antibiotic. It was suggested that the establishment of large populations of *Agrobacterium* carrying plasmid pAgK84 could threaten the effectiveness of *A. radiobacter* K84 as a biocontrol agent (Stockwell et al., 1996). To minimize the risk of transfer of this plasmid by *A. tumefaciens*, a derivative strain of K84 was constructed (K1026), that still produces agrocin 84 but lacks the region necessary for the transfer of the plasmid pAgK84. The judicious use of biocontrol agents and particularly of the new strain K1026 should minimize the risk of pAgK84 transfer into pathogenic *Agrobacterium* strains and thus help to preserve the effectiveness of *A. radiobacter* as a biocontrol agent against crown gall.

### Resistance to Cell Wall Degrading Enzymes Produced by Biocontrol Agents

Most biocontrol agents acting by hyperparasitism produce enzymes such as chitinases and glucanases. Specific mechanisms can help plant pathogens to protect themselves against these cell wall degrading enzymes. For example, the synthesis of melanin polymers could help microorganisms to protect against microbial enzymes (Bell and Wheeler, 1986). The formation of chlamydospore-like structures and vacuolated portions in mycelium allows the pigeonpea wilt pathogen *Fusarium udum* to maintain a slow growth and the production of conidia in presence of the hyperparasitic bacterium *Bacillus subtilis* AF1 (Harish et al., 1998). Another possible mechanism implies the repression of the synthesis of enzymes produced by the biocontrol agent. For instance, the mycotoxin DON, produced by *Fusarium culmorum* and *Fusarium graminearum* represses the expression of genes coding for chitinases in the biocontrol strain *Trichoderma atroviride* P1 (Lutz et al., 2003).

### Resistance to Defense Compounds Produced by the Plants

The induction of resistance in plants involves the synthesis and the activation of plant defense compounds as the result of activity of an elicitor. This induction leads to rapid production of reactive oxygen species, modifications of cuticle thickness, cell wall appositions, accumulation of phenolic compounds and production of phytoalexins and pathogenesis-related proteins rendering the plants more resistance to pathogen attack (Wiesel et al., 2014). To survive and become established in a resistance-induced plant, plant pathogens have to overcome these induced host defense chemicals. Plant pathogens may evolve a combination of strategies to colonize and survive in this environment. Such strategies could include transporting toxic chemicals out of the cell or sequestrating them in cellular organelles, detoxifying host defense compounds by converting or modifying them and interfering with host signaling (Morrissey and Osbourn, 1999).

#### Excretion

A way for plant pathogens to cope with host defense chemicals is to secrete them out of the cells as in the case of antimicrobial compounds produced by biocontrol agents (see section above). For example, ABC transporter activity has been implicated in the tolerance of *Nectria haematococca* to pisatin, a phytoalexine of *Pisum sativum* (Denny et al., 1987), of *B. cinerea* to resveratrol, a phytoalexin of grape (Schoonbeek et al., 2001) and of *Gibberella pulicaris* to rishitin, a trait that is required for virulence of the fungus on potato tubers (Fleissner et al., 2002). Similarly, a recent study on *Grosmannia clavigera*, a fungal pathogen of pine trees, showed that this fungus can survive and become established in pine tissues by the induced expression of an efflux ABC transporter GcABC-G1 (Wang et al., 2013). This allows the fungus to cope with intracellular levels of monoterpenes, which are among the most abundant antimicrobial pine defense chemicals. ABC transporters are potentially involved in the virulence and aggressiveness of fungal plant pathogens, by decreasing the toxic effect of phytoalexins (Del Sorbo et al., 2000).

#### Metabolization

*Botrytis cinerea* is for instance able to detoxify the alpha-tomatine, a phytoanticipin found in tomato plants (Quidde et al., 1998). In this study, 12 out of 13 isolates of *B. cinerea* tested are able to degrade alpha-tomatine thanks to a xylase activity that prevents the saponin from complexing with the fungus membrane and therefore suppresses its antifungal activity. The ability of *B. cinerea* to overcome the phytoalexin resveratrol, which synthesis is induced in grapevine, has also been observed (Sbaghi et al., 1996). A correlation between the ability of eight isolates of *B. cinerea* to detoxify the resveratrol and their pathogenicity on grapevine leaves was shown. Some plant pathogens, like the fungus *Microcyclus ulei*, are tolerant to hydrocyanic acid, a volatile compound produced by plants in response to various damages (Osbourn, 1996). This tolerance might be due to cyanide-resistant respiration. Other fungi could also detoxify this compound by converting it to formamide, *via* cyanide-hydratases. Some plant pathogens are then able to degrade defense compounds and thus might be able to overcome the effect of biocontrol agents acting through plant defense induction.

#### Protection Against Oxidative Damage

The hypersensitive reaction (HR) involves the production of reactive oxygen species that leads to cell death and it is generally consider as a signal to induce the synthesis of antimicrobial compounds like phytoalexins. It usually prevents the fungal penetration into host tissue but it has been shown that HR facilitates the colonization of the plants by necrotroph fungal pathogens like *B. cinerea* or *S. sclerotiorum* (Govrin and Levine, 2000; Govrin et al., 2006) or *Rhizoctonia solani* (Shibu et al., 2012). These plant pathogenic fungi might therefore exploit particular defense mechanisms of the plant to overcome the induced resistance effect of a biocontrol agent. Moreover, many other plant pathogens can protect themselves against oxidative damage (Duffy et al., 2003).

### Reducing Populations of Biocontrol Agents

Some plant pathogens can significantly slow down the growth of biocontrol agents by exploiting more rapidly the resources within their environment and then affecting their efficacy. This was shown in a study conducted to evaluate the effect of several fungal root pathogens on the capacity of biocontrol strains *P. fluorescens* 2–79 and Q72a80 to persist in the wheat rhizosphere (Mazzola and Cook, 1991). For instance, in presence of some *Pythium* species, the population of *P. fluorescens* declines. In this case, the infection by *Pythium* tends to reduce the root surface available for bacterial colonization which, as a consequence, tends to limit the population of potential antagonists. In another study, the plant pathogenic oomycete *P. ultimum* can affect the genes expression of the biocontrol agent *P. fluorescens* F113, leading to the decline of its population (Fedi et al., 1997). This population size reduction limits the competition in the rhizosphere for nutrients leaking from wounds caused by *P. ultimum* on sugar beet roots. Competition may also occur on the phyllosphere where the microbial community is also important in number and diversity and where microorganism–microorganism interactions can occur (Vorholt, 2012). This microbial community may also affect the efficacy of biocontrol agents. Knowledge about plant microbiome (rhizosphere, phyllosphere, endosphere) and the microbial interactions occurring on those niches should contribute to the improvement of biological control.

## Conclusion

Despite the limited number of published studies dedicated to this topic, it is clear from this review that plant pathogens can display various (including very low) levels of sensitivity to biocontrol agents, regardless of the complexity of their mode of action. Certain pathogens also have the potential to adapt in a few generation to the selection pressure exerted by biocontrol agents. Available information is sufficient to suggest that the assumption that durability of biological control is necessarily higher than that of chemical control may not always be justified. However, it is not sufficient to draw conclusions about the existence of specific traits related to the plant pathogen or related to the biocontrol agent that could explain the loss of effectiveness of a biocontrol agent in practice.

There is much need for further studies on issues related to this topic. Among them, the establishment of baseline sensitivity (i.e., testing a sufficient number of samples of field populations of target plant pathogens for their degree of sensitivity to one or more biocontrol agents) would be helpful in future surveys of biocontrol agents. Such monitoring is carried out routinely for fungicide resistance in fungal plant pathogens world-wide (Brent and Hollomon, 2007). It is a first step to evaluate the distribution and the potential impact of resistance in the field. Moreover, detection of partially resistant isolates may indicate a risk of developing a more severe resistance that subsequently may initiate a possible loss of control in the field. Monitoring can be done at specific sites, for example to ascertain the implication of resistance in cases when loss of biocontrol effectiveness is observed in the field. It can also be done at different dates to document the evolution of sensitivity following series of treatments with a given biocontrol agent and to monitor possible shifts in the natural populations of a plant pathogen, as suggested by Yang et al. (2008). Finally, cross-resistance with other existing biocontrol agents (or possibly fungicides) may also be determined.

In addition to the population approach described above, experimental evolution studies are also needed to evaluate the ability of plant pathogens to evolve under the selection pressure exerted by a biocontrol agent. This approach will result in identifying risk factors that can foster the selection of strains of plant pathogens resistant to biocontrol agents. Various biocontrol agents and different plant pathogens must be tested in the future. This will result in identifying types of biocontrol agents with lower risk of efficacy loss, i.e., modes of action of biocontrol agents that does not favor the selection of resistant isolates in natural populations of plant pathogens. Even though all biocontrol agents should create selection pressure on target populations of plant pathogen, some modes of action may present a clear opportunity for pathogens to evolve resistance. For example, a mechanism involving antibiosis would, by analogy with the fungicides, be considered a high risk to be overcome, whereas a multiple or a more complex mode of action would indicate relatively low risk. This knowledge is essential to ensure a durable efficacy of biocontrol agents on target plant pathogens.

Even if the data are too sparse to suggest general statement on the use of biocontrol agents in practice, this review highlights the necessity for careful management of their use once they become commercially available in order to avoid repeating the mistakes made with chemical fungicides. For instance, we should consider alternating or combining biocontrol agents with different mechanisms of action. In addition to possibly ensure the durability of biological control, the combination of several biocontrol agents have shown to improve the efficacy and reduce the variability of efficacy (Flaherty et al., 2000; Guetsky et al., 2001, 2002; de Boer et al., 2003). The context of integrated pest management is probably an effective way to reduce the risk of resistance development but it necessitates to evaluate the compatibility of the biocontrol agents together with other protection methods. The combined use of biocontrol agents together with pesticides requires that their efficiency is not altered when applied in conjunction or in alternation.

Significant research efforts are also needed to anticipate the potential failure of biological control and integrate durability concerns in the screening procedure of new biocontrol agents. According to the results mentioned in this review, caution should be applied when screening and selecting isolates of biocontrol agents, to ensure a wide representation of the targeted plant pathogen population. To test the efficacy of potential biocontrol agents or to screen for new biocontrol agents against plant diseases, most studies continue to use a single isolate or relatively few isolates. Innovative screening procedures based on the known mode of action have already been developed (Schoonbeek et al., 2007; Sansone et al., 2011). We must go further by incorporating in the screening procedure, knowledge on the ability of plant pathogen to counteract these modes of action.

### Conflict of Interest Statement

The authors declare that the research was conducted in the absence of any commercial or financial relationships that could be construed as a potential conflict of interest.
